# Nomogram to predict severe *Mycoplasma pneumoniae* pneumonia in children

**DOI:** 10.3389/fped.2026.1735063

**Published:** 2026-03-04

**Authors:** Yuan Zhang, Jie Min, Liang Gong

**Affiliations:** Department of Respiratory Medicine, Xuzhou Children’s Hospital, Xuzhou, Jiangsu, China

**Keywords:** *mycoplasma pneumoniae* pneumonia, nomogram, prediction model, risk factors, severe *mycoplasma pneumoniae* pneumonia

## Abstract

**Background:**

*Mycoplasma pneumoniae* pneumonia (MPP) is a prevalent community-acquired pneumonia in children, and severe MPP (SMPP) poses a prominent threat to pediatric health with rapid progression, high complication rates, and increased clinical management burden. Clinically, the capacity to identify children at high risk of SMPP remains inadequate. The aim of this study was to develop and validate a nomogram for predicting SMPP in children with MPP.

**Methods:**

A total of 475 children with MPP admitted to Xuzhou Children’s Hospital from Jan. 2023 to Dec. 2024 were enrolled, meeting specific inclusion/exclusion criteria. They were categorized into severe MPP (SMPP, *n* = 151) and non-SMPP (*n* = 324) groups, then randomly split into training (*n* = 332) and validation (*n* = 143) cohorts at a 7:3 ratio. Demographic, clinical, laboratory data and derived inflammatory indicators were collected. LASSO and multivariate logistic regression were used to construct a nomogram, with ROC, calibration curves and DCA for evaluation. The study was ethically approved.

**Results:**

Using LASSO and multivariate logistic regression analyses, fever duration (OR = 1.271, *P* < 0.0001), red blood cell count (OR = 0.300, *P* = 0.0069) and albumin (OR = 0.795, *P* = 0.0002) were identified as independent predictors. The nomogram showed good discrimination (training cohort AUC=0.8574, 95%CI:0.8162–0.8986; validation cohort AUC=0.8147, 95%CI:0.7435–0.8859). The Hosmer-Lemeshow test yielded *P* = 0.551 in the training set and *P* = 0.553 in the validation set, and calibration curves in both cohorts confirmed excellent model fit, while DCA verified substantial clinical utility, supporting the nomogram’s clinical value in pediatric SMPP prediction

**Conclusion:**

We developed and validated a practical, user-friendly nomogram for predicting SMPP in children with MPP, which could facilitate early identification and risk stratification of SMPP.

## 1 Introduction

*Mycoplasma pneumoniae* (*M. pneumoniae*) is one of the primary causes of community-acquired pneumonia (CAP) in the pediatric population (1, 2). Most cases of *M. pneumoniae* pneumonia (MPP) in children present with mild symptoms and without hospitalization. However, a handful of pediatric patients progress to severe MPP (SMPP), and epidemiological surveys have documented a gradual increase in the incidence of SMPP in recent years (3, 4). SMPP reflects the high severity of MPP and meets the diagnostic criteria for severe CAP (5). Notably, SMPP is prone to potentially fatal conditions, including myocarditis, nephritis, encephalitis and hemolytic anemia (6), highlighting the urgency of early and accurate identification of high-risk children to enable timely intervention and reduce mortality.

To date, several predictive tools for pediatric SMPP have been developed, including machine learning models and nomograms (7–11). Nevertheless, these existing models have significant limitations: some are established based on small sample data and fail to clearly distinguish differences in disease occurrence among children of different age groups, resulting in limited data representativeness; others focus solely on inflammatory markers or coagulation function indicators, lacking integration of multi-system parameters; and still others regard lung auscultation as a key predictive factor, but different physicians may yield inconsistent assessment results for the same case, which in turn undermines the model's stability. In contrast, a nomogram integrated with multiple accessible objective indicators can comprehensively weigh the predictive value of each factor, thereby improving the accuracy and practicability of early SMPP identification. Therefore, in this retrospective study, we developed and validated a nomogram-based diagnostic prediction model for pediatric SMPP, aiming to enhance the practicality and accuracy of early SMPP identification and ultimately reduce the occurrence of adverse clinical outcomes.

## 2 Materials and methods

### 2.1 Patients

This study gathered clinical data from 475 MPP children at Xuzhou Children's Hospital between January 2023 and December 2024. The criteria for inclusion were: (a) Aged 1–14 years; (b) Meeting clinical diagnostic criteria for pneumonia; (c) Positive for MP- DNA in nasopharyngeal secretions via real-time quantitative PCR and positive serology via chemiluminescent immunoassay (3); (d) Complete and accessible clinical data and laboratory test results.

The criteria for exclusion were: (a) History of recurrent respiratory infections; (b) Severe organic diseases or history of major surgery; (c) Evidence of coinfection with other pathogens; (d) Incomplete clinical data.

SMPP was defined based on meeting the diagnostic criteria (5): (a) Poor general condition; (b) Consciousness disturbance, cyanosis, or respiratory dysfunction; (c) Hypoxemia, assisted breathing, intermittent apnea, or oxygen saturation < 92%; (d) Persistent hyperpyrexia lasting more than 5 days, or ultrahyperpyrexia; (e) Dehydration or food refusal; (f) Chest imaging showing the following findings: unilateral lung infiltration ≥ 2/3, multilobar lung infiltration, pleural effusion, pneumothorax, atelectasis, lung necrosis, or lung abscess; (g) Presence of extrapulmonary complications. Eligible patients were categorized into the SMPP group (*n* = 151) and non-SMPP group (*n* = 324). Subsequently, the total cohort (*n* = 475) was randomly divided into a training cohort (70%, *n* = 332) and a validation cohort (30%, *n* = 143) at a 7:3 ratio.

### 2.2 Data collection

The independent variables collected from MPP children were as follows: (a) Demographic characteristics: Sex, age; (b) Clinical indicators: Duration of fever—recorded within 24 h of admission, defined as the interval from first fever onset to hospital admission; (c) Laboratory parameters: White blood cell count (WBC), neutrophil ratio (N%), lymphocyte ratio (L%), monocyte ratio (M%), eosinophil ratio (E%), basophil ratio (B%), neutrophil count, lymphocyte count, monocyte count, eosinophil count, red blood cell count (RBC), hemoglobin (HGB), platelet count (PLT), C-reactive protein (CRP), lactate dehydrogenase (LDH), alanine aminotransferase (ALT), albumin (ALB), D-dimer; (d) Derived inflammatory indicators: neutrophil-to-lymphocyte ratio (NLR), platelet-to-lymphocyte ratio (PLR), systemic immune inflammation index (SII), systemic inflammation response index (SIRI).

### 2.3 Statistical analysis

Statistical analyses were conducted using SPSS 27.0 and R 4.2.1 software, with *P* < 0.05 indicating statistical significance. Quantitative data were presented as the mean ± standard deviation (SD) or median (25th–75th percentile), and categorical data were presented as counts and percentages. Intergroup comparisons were analyzed via t-tests, Wilcoxon rank-sum tests, or Pearson's chi-square tests.

**Figure 1:**
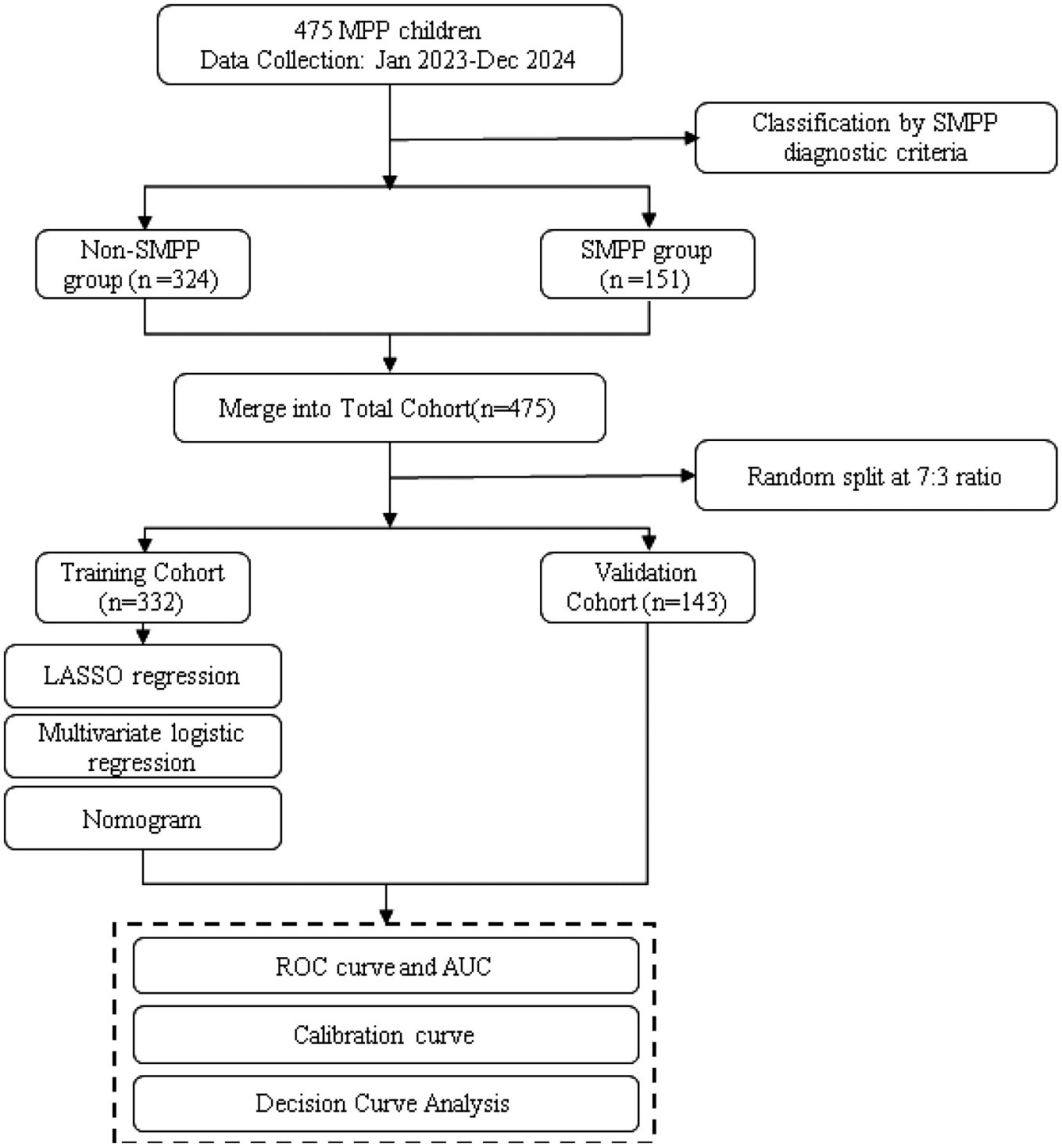
Flowchart of this study.

LASSO regression analysis was used to screen potential predictive factors, and multivariate logistic regression analysis was applied to identify independent risk factors for SMPP and construct a nomogram. The receiver operating characteristic (ROC) curve and area under the curve (AUC) were used to evaluate the model's discriminatory ability. Calibration curves were used to assess the consistency between predicted and actual outcomes, and decision curve analysis (DCA) was performed to verify the clinical utility of the nomogram (12). A detailed flowchart is shown in Figure 1.

## 3 Results

### 3.1 Patient characteristics

The characteristics of all 475 enrolled children are summarized in Table 1. Among the participants, 31.8% (151/475) were diagnosed with SMPP, with a median age of 6.79 [interquartile range (IQR) 5–8] years. There were no statistically significant differences in sex or age between the SMPP and non-SMPP groups (both *P* > 0.05). The SMPP group had a significantly longer duration of fever than the non-SMPP group (median: 9.49 vs. 6.33 days, *P* < 0.001). Additionally, significant intergroup differences were observed in multiple laboratory parameters, including WBC, N%, L%, neutrophil count, lymphocyte count, monocyte count, RBC, HGB, CRP, LDH, ALT, ALB, D-dimer, NLR, PLR and SII (all *P* < 0.05). As depicted in Table 2, there were no significant differences in demographic and clinical characteristics between the training set and validation set (*P* > 0.05).

**Table 1:** Baseline characteristics between the Non-SMPP and SMPP group.

Variable	Non-SMPP group (*n* = 324)	SMPP group (*n* = 151)	*P*-value
Sex (male), %	155 (47.8)	77 (51.0)	0.588
Age, years	6.32 (5,8)	6.79 (5,8)	0.076
Duration of fever, days	6.33 (5,8)	9.49 (7,11)	<0.001
WBC count, ×10^9^/L	9.55 (6.47,11.25)	10.83 (7.78,12.39)	0.006
Neutrophil ratio, %	61.86 (53.85,71.45)	69.34 (60.80,78.3)	<0.001
Lymphocyte ratio, %	29.40 (20.35,36.55)	21.76 (13.70,28.60)	<0.001
Monocyte ratio, %	7.23 (5.75,8.5)	7.23 (5.55,8.50)	0.976
Eosinophil ratio, %	1.33 (0.20,1.6)	1.52 (0.20,2.10)	0.337
Basophil ratio, %	0.18 (0.10,0.20)	0.16 (0.00,0.20)	0.186
Neutrophil count, ×10^9^/L	6.06 (3.66,7.46)	7.59 (4.81,9.09)	<0.001
Lymphocyte count, ×10^9^/L	2.68 (1.70,3.09)	2.27 (1.35,2.85)	0.004
Monocyte count, ×10^9^/L	0.67 (0.44,0.83)	0.79 (0.45,0.95)	0.007
Eosinophil count, ×10^9^/L	0.12 (0.02,0.16)	0.16 (0.01,0.23)	0.092
RBC count, ×10^12^	4.52 ± 0.37	4.38 ± 0.36	<0.001
HGB, g/L	125.63 ± 9.78	123.44 ± 8.82	0.02
PLT count, ×10^9^/L	319.40 (243,384)	326.79 (262.5,383)	0.132
CRP, mg/L	6.40 (0.01,7.79)	14.05 (0.01,17.95)	<0.001
LDH, U/L	355.95 (276.5,400)	507.92 (348.5,594)	<0.001
ALT, U/L	22.02 (11,20)	49.55 (14.5,40.5)	<0.001
ALB, g/L	41.38 ± 2.75	37.89 ± 3.30	<0.001
D-dimer, mg/L	1.07 (0.53,1.12)	3.34 (1.20,3.32)	<0.001
NLR	2.33 (1,3)	3.89 (2,5)	<0.001
PLR	143.44 (92,172)	182.13 (114,221)	<0.001
SII	749.54 (290,950)	1,210.87 (505,1585)	<0.001
SIRI	1.55 (0.53,1.87)	2.95 (0.95,3.70)	<0.001

WBC, white blood cell; RBC, red blood cell; HGB, hemoglobin; PLT, platelet; CRP, C-reactive protein; LDH, lactate dehydrogenase; ALT, alanine aminotransferase; ALB, albumin.

**Table 2:** Baseline characteristics in training and validation set.

Variable	Training Set (*n* = 332)	Validation Set (*n* = 143)	*P*-value
Sex			0.690
Female	172 (51.8)	71 (49.7)	
Male	160 (48.2)	72 (50.3)	
Age, years	6.40 (2.55)	6.62 (2.54)	0.381
WBC count, ×10^9^/L	9.95 (4.84)	9.97 (4.46)	0.959
Neutrophil ratio, %	63.63 (13.23)	65.65 (13.05)	0.125
Lymphocyte ratio, %	27.41 (12.19)	25.95 (11.73)	0.225
Monocyte ratio, %	7.33 (2.52)	7.02 (2.65)	0.234
Eosinophil ratio, %	1.47 (1.86)	1.21 (1.60)	0.158
Basophil ratio, %	0.17 (0.14)	0.17 (0.14)	0.951
Neutrophil count, ×10^9^/L	6.47 (3.77)	6.75 (3.81)	0.457
Lymphocyte count, ×10^9^/L	2.61 (1.67)	2.41 (1.32)	0.214
Monocyte count, ×10^9^/L	0.72 (0.40)	0.68 (0.39)	0.432
Eosinophil count, ×10^9^/L	0.14 (0.19)	0.11 (0.16)	0.092
RBC count, ×10^12^	4.48 (0.37)	4.47 (0.37)	0.790
HGB, g/L	125.17 (9.44)	124.36 (9.75)	0.395
PLT count, ×10^9^/L	324.40 (107.31)	315.60 (101.35)	0.405
CRP, mg/L	8.59 (18.97)	9.39 (22.84)	0.692
LDH, U/L	398.18 (169.75)	418.38 (204.37)	0.265
ALT, U/L	26.35 (50.94)	41.06 (114.84)	0.054
ALB, g/L	40.46 (3.29)	39.82 (3.48)	0.056
D-dimer, mg/L	1.63 (2.30)	2.17 (3.60)	0.052
NLR	2.70 (2.62)	3.10 (2.94)	0.139
PLR	154.92 (83.85)	157.65 (78.05)	0.739
SII	867.26 (868.62)	963.36 (952.72)	0.283
SIRI	1.93 (2.37)	2.14 (2.53)	0.390

WBC, white blood cell; RBC, red blood cell; HGB, hemoglobin; PLT, platelet; CRP, C-reactive protein; LDH, lactate dehydrogenase; ALT, alanine aminotransferase; ALB, albumin.

### 3.2 Independent risk factors for the model

**Figure 2:**
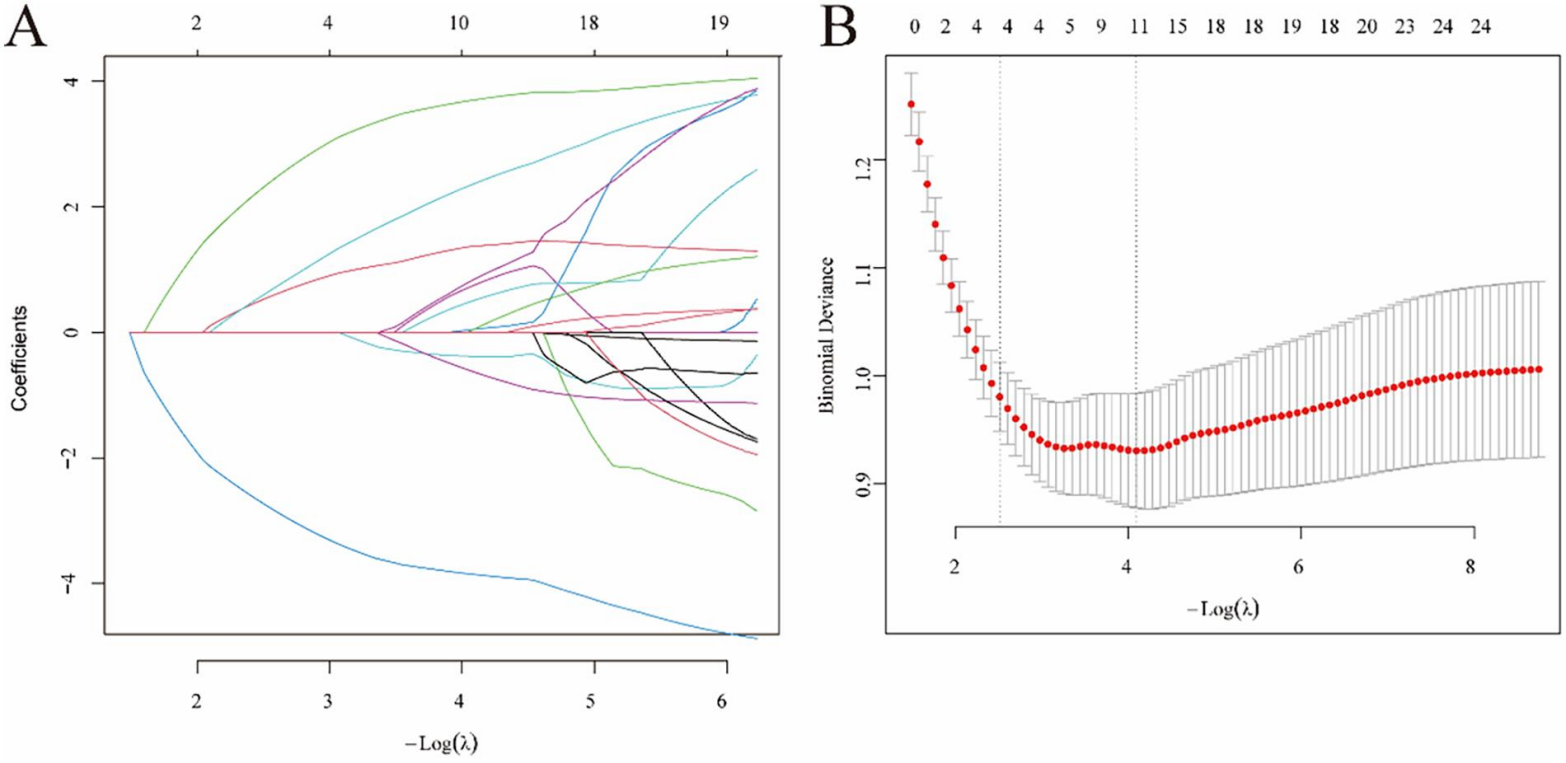
Variable selection was conducted via least absolute shrinkage and selection operator (LASSO) logistic regression. **(A)**LASSO coefﬁcient proﬁles of 26 clinical features. **(B)** The optimal parameter [*λ*] in the LASSO model was performed using the minimum criterion method with tenfold cross-validation. The optimal value of *λ* is indicated by the vertical dashed line. Among these values, *λ* = 0.0166, corresponding to a logarithm of *λ* equal to −0.898, was selected as the optimal choice, which identified 11 features with nonzero coefficients.

LASSO regression analysis was conducted on the initial 26 variables collected from pediatric patients, and 11 potential risk factors for SMPP were identified (Figure 2), including duration of fever, L%, M%, monocyte count, eosinophil count, RBC, LDH, ALT, ALB, D-dimer, and PLR. Subsequently, multivariate logistic regression analysis was performed on these 11 candidate predictors. The results showed that duration of fever [OR = 1.271, 95% confidence interval (95% CI): 1.129–1.431, *P* < 0.0001], RBC (OR = 0.300, 95% CI: 0.123–0.717, *P* = 0.0069), and ALB (OR = 0.795, 95% CI: 0.703–0.897, *P* = 0.0002) were independent predictors of SMPP. The results of the multivariate analysis for SMPP in the training set are presented in Table 3.

**Table 3:** Risk factors selected after multivariate logistic regression analysis.

Variable	*P*-value	Odds Ratio (OR)	95% Confidence Interval (95% CI)
Duration of fever	<0.0001	1.2710	1.1290–1.4310
Lymphocyte ratio	0.5282	1.0128	0.9735–1.0537
Monocyte ratio	0.6380	1.0373	0.8920–1.2070
Monocyte count	0.1862	2.0660	0.7390–5.7790
Eosinophil count	0.5714	1.6250	0.2930–9.0220
RBC	0.0069	0.2990	0.1240–0.7170
LDH	0.3882	1.0010	0.9987–1.0033
ALT	0.4024	1.0018	0.9975–1.0061
ALB	0.0002	0.7940	0.7040–0.8980
D-dimer	0.0819	1.1750	0.9810–1.4070
PLR	0.3730	1.0025	0.9968–1.0082

RBC, red blood cell; LDH, lactate dehydrogenase; ALT, alanine aminotransferase; ALB, albumin.

### 3.3 Development and validation of the nomogram

**Figure 3:**
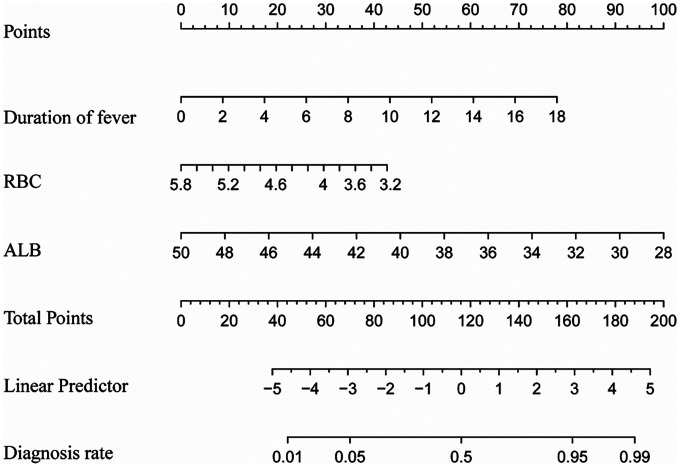
Nomogram incorporating duration of fever, red blood cell (RBC) count, and albumin (ALB) predicts the probability of developing severe *Mycoplasma pneumoniae* pneumonia (SMPP).

**Figure 4:**
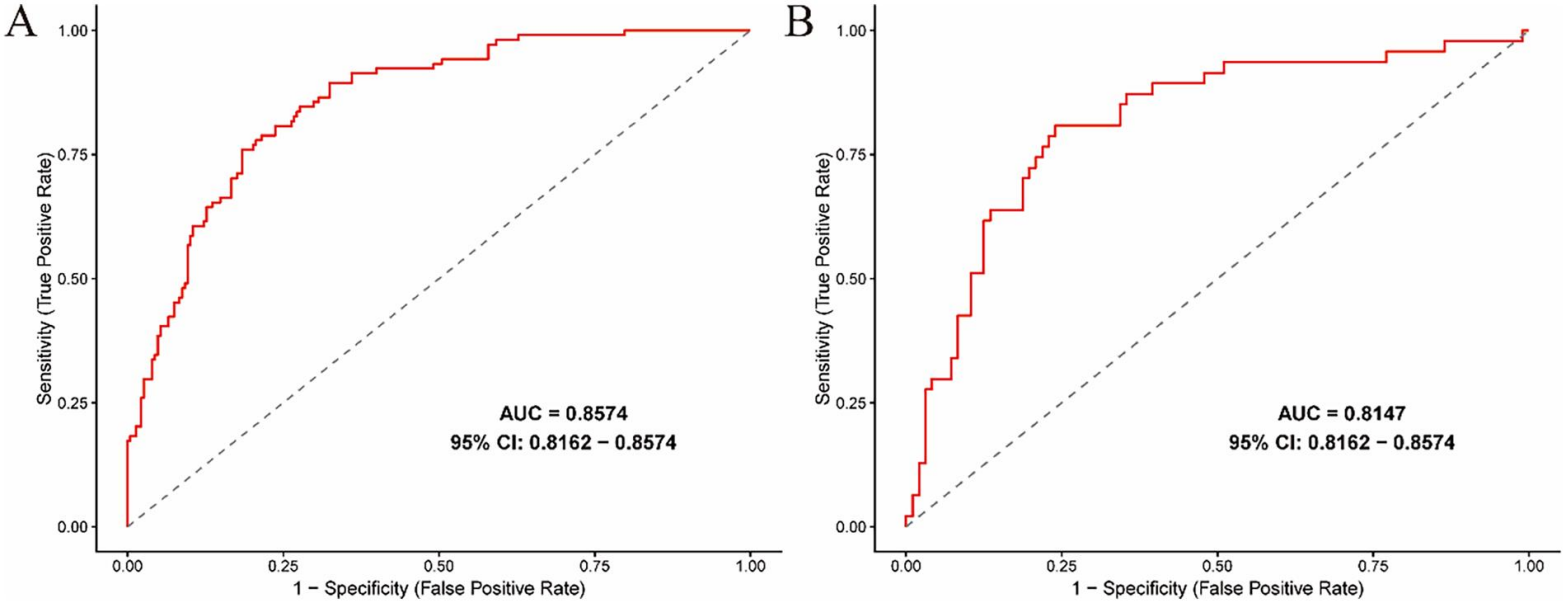
The area under the receiver operating characteristic curve [AUC] for the discrimination of the model. **(A)** The training set, 0.8574 [95% CI: 0.8162−0.8574]. **(B)** The validation set, 0.8147 [95% CI: 0.8162−0.8574].

**Figure 5:**
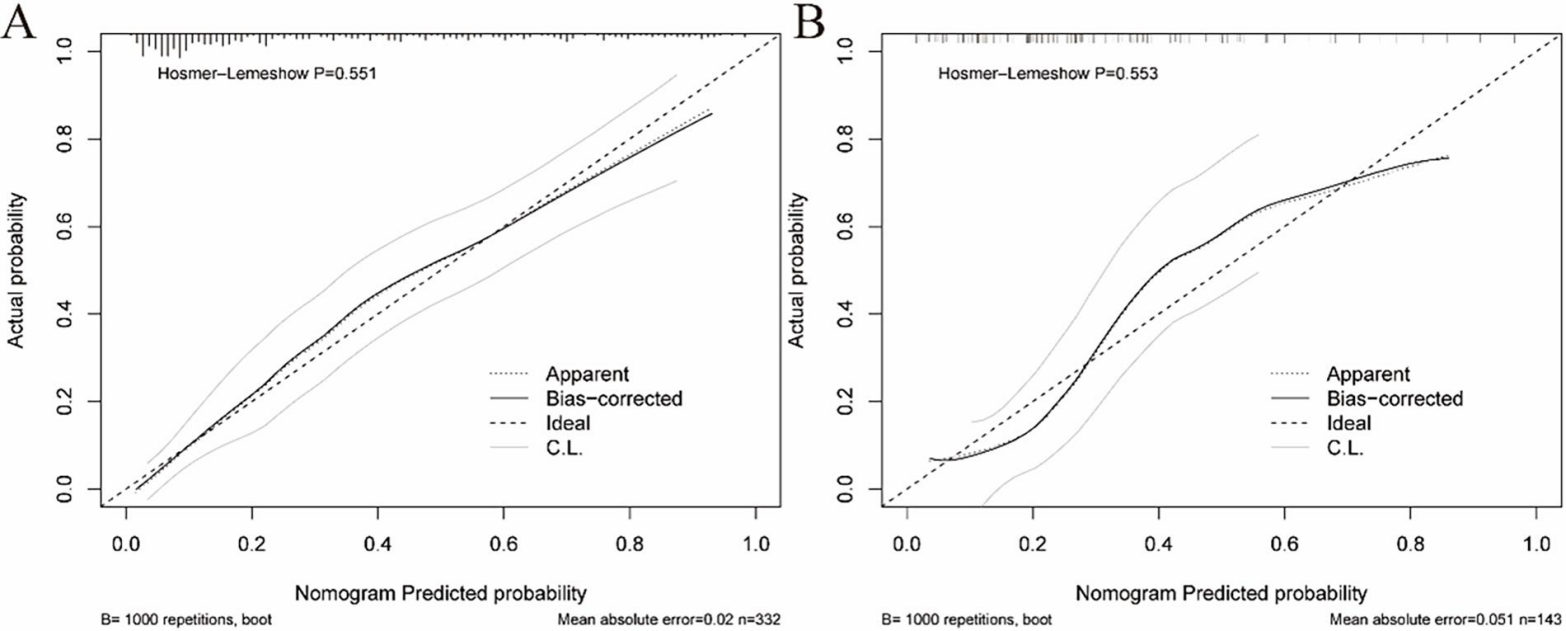
Calibration curves of the predictive SMPP risk nomogram at the training **(A)** and validation cohorts **(B)** All *P*-value >0.05 in the hosmer–lemeshow test suggested an agreement between the predicted probabilities and observed outcomes.

**Figure 6:**
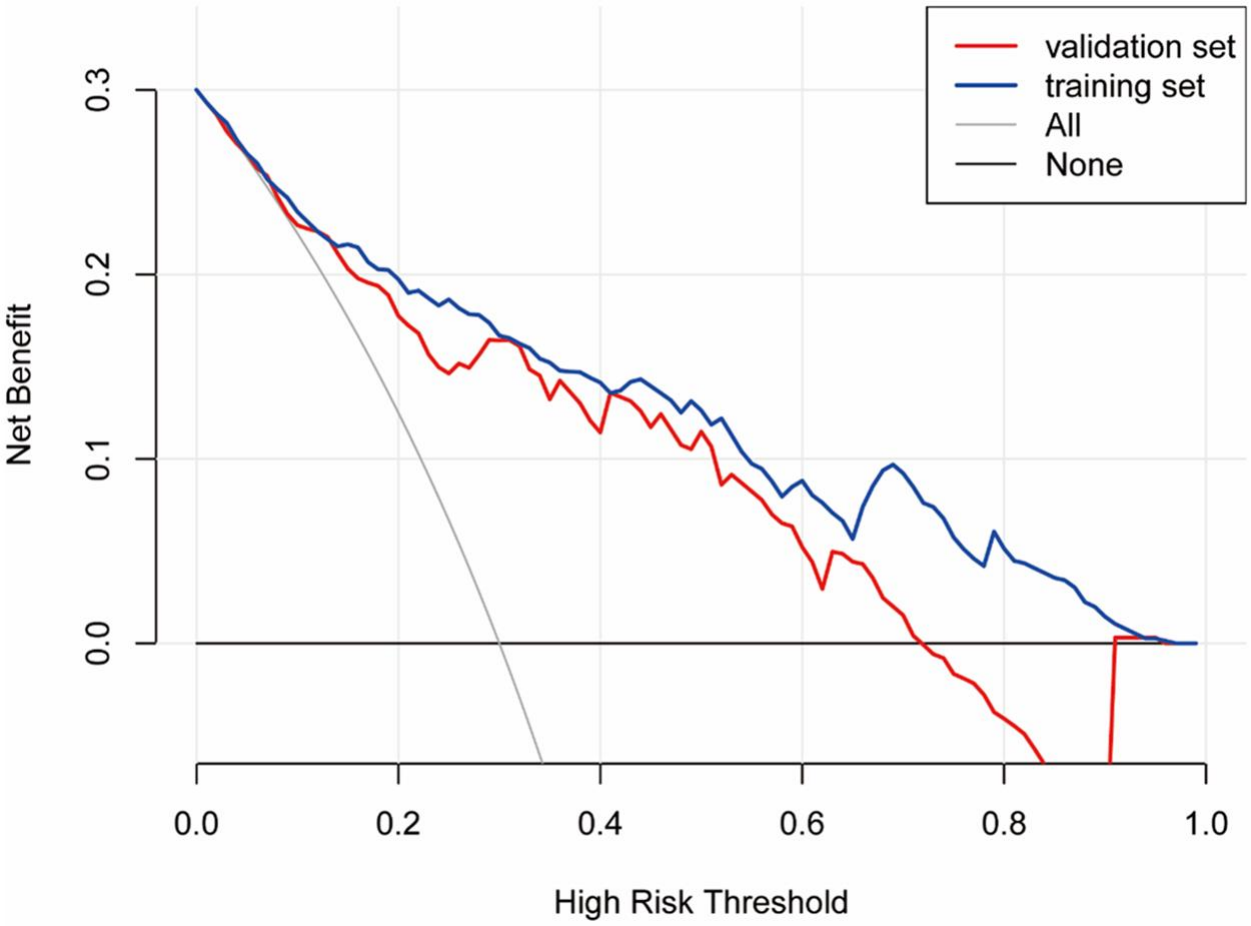
Decision curve analysis (DCA) for the SMPP nomogram. The analysis was conducted on both the training set and the validation set.

A nomogram for predicting SMPP was constructed using the identified independent predictors. As shown in Figure 3, three independent variables, duration of fever, RBC, and ALB, were included to develop the model. The risk of SMPP was positively correlated with the total score, which was the sum of the points allocated to each factor in the nomogram. The AUC in the training cohort was 0.8574, with a 95% confidence interval of 0.8162–0.8574, whereas the validation cohort showed an AUC of 0.8147, with a 95% confidence interval of 0.8162–0.8574 (Figure 4). In both the training set (Hosmer-Lemeshow *P* = 0.551) and validation set (Hosmer-Lemeshow *P* = 0.553), the calibration curves (apparent and bias-corrected) were fairly close to the ideal line, indicating a good agreement between the predicted probabilities and actual observations (Figure 5). Decision Curve Analysis (DCA) curves demonstrated that the nomogram offered substantial clinical utility, with significant advantages over non-clinical strategies (Figure 6).

## 4 Discussion

*M. pneumoniae* has long been a major pathogenic cause of CAP in children and adolescents (8, 13). Most MPP cases are mild, with only a small minority presenting as the severe form (SMPP); however, the overall proportion of SMPP has been on the rise in recent years (3, 14). Among 494 children with MPP admitted to the specialized ward of Children's Hospital of Fudan University between August and September 2023, as many as 124 cases (25.1%) were diagnosed with SMPP (14). Xu et al. reported that 46.6% of pediatric MPP inpatients (173/371) in Wuhan from 2020 to 2022 presented with severe clinical manifestations (15), and another study indicated that 50.6% of MPP cases were severe and complicated with more extrapulmonary abnormalities (16). In the present study, the proportion of SMPP among enrolled patients was 31.8% (151 cases). The observed discrepancies in SMPP incidence across these studies may be attributed to three main factors: first, variations in study populations and sample sizes; second, regional differences in epidemiological and clinical characteristics of MPP; third, the lack of a unified and standardized diagnostic criteria for severe Mycoplasma pneumoniae pneumonia to date.

Severe MPP is characterized by severe pulmonary involvement and multiple extrapulmonary complications (6). Pediatric patients are particularly prone to SMPP progression, attributable to nonspecific clinical manifestations in the early stage, immature immune systems, and underdeveloped tissue and organ function (2, 17). Children with SMPP face a high risk of developing necrotizing pneumonitis, myocarditis, hemolytic anemia, acute pancreatitis, and a wide array of other extrapulmonary complications (6), which poses a substantial threat to pediatric health. Accordingly, early prediction of SMPP can assist clinicians in the rational allocation of critical care resources and timely identification of high-risk patients for prompt diagnosis and intervention, thereby enabling more targeted and optimized clinical management. We created and validated a nomogram in this study to predict SMPP in children with MPP. The nomogram incorporated 3 variables, including duration of fever, RBC count and ALB level. The ROC, DCA, and calibration curves all revealed that the nomogram possesses good discriminatory power, accurate calibration, and clinical usefulness. Collectively, our study findings may contribute to the advancement of early screening and predictive assessment for SMPP in pediatric clinical practice.

Previous studies have confirmed that fever characteristics are closely associated with the progression of SMPP (18)A study by Ken B et al. clarified that both the intensity (high-grade fever ≥39.0 °C) and duration (≥8 days) of fever can serve as early screening indicators for SMPP, and the fever characteristics of severe cases are closely correlated with elevated inflammatory factors such as CRP and LDH (19). Furthermore, another study verified that preadmission fever duration is an independent risk factor for necrotizing pneumonia (NP) and refractory MPP (RMPP), and prolonged fever directly indicates an increased risk of severe disease (13). Notably, a recent multicenter study in South Korea further validated that patients with lobular/lobar consolidation, a core radiological marker for MPP severity, had significantly longer fever duration (median 8 days vs. 7 days, *P* = 0.020) and a higher proportion of high-grade fever (71.0% vs. 60.1%, *P* = 0.034) (20). These findings are highly consistent with the data of the present study: the median fever duration was 6.33 days in the non-SMPP group and 9.49 days in the SMPP group. Importantly, our fever duration was recorded within 24 h of admission (defined as the interval from first fever onset to admission), making it an early accessible indicator—this aligns with Lee et al.'s validation of pre-admission fever duration as an early SMPP predictor (20). Moreover, our multivariate analyses identified fever duration as an independent risk factor for the progression of pediatric MPP to SMPP, a relationship that may be linked to the excessive inflammatory response and severe lung injury induced by *M. pneumoniae* infection.

During *M. pneumoniae* infection, the RBC count tends to decrease, and a few patients may develop significant anemia (21–23). MP infection stimulates the body to produce polyclonal IgM-type cold agglutinins, which specifically recognize the I antigen on the surface of red blood cells, activate complement, and ultimately lead to red blood cell destruction through extravascular hemolysis (23). In our study, the RBC count of children with SMPP was significantly lower than that of children with non-SMPP (4.38 ± 0.36 × 10¹²/L vs. 4.52 ± 0.37 × 10¹²/L, *P* < 0.001). This observation may be attributed to the excessive systemic inflammatory response triggered by SMPP, which exacerbates immune-mediated hemolysis and thereby further reduces the RBC count (24). In addition, in our prediction model, RBC count was included as an independent risk factor for the first time, indicating that RBC count is a useful marker for predicting the severity of MPP in children.

Albumin is the most abundant plasma protein. Under inflammatory conditions, increased capillary permeability leads to massive leakage of albumin into the interstitium, coupled with enhanced albumin degradation, thereby resulting in hypoalbuminemia. Albumin is negatively correlated with the degree of inflammation, lower levels indicate more severe inflammation (25). Our study showed that there was a significant difference in serum albumin levels between children with SMPP and those with non-SMPP: the serum albumin level in the SMPP group was 37.89 ± 3.30 g/L, which was significantly lower than that in the non-SMPP group (41.38 ± 2.75 g/L), with a statistically significant difference (*P* < 0.001). This finding is consistent with previous studies (24, 26, 27). Additionally, albumin, either alone or as a component of composite indicators, has been proven to be an effective predictor of disease severity and mortality in conditions such as sepsis and Kawasaki disease (28, 29). In the present study, albumin was an independent risk factor for SMPP in children (OR = 0.795, 95% CI: 0.704–0.898, *P* = 0.0002), with lower albumin levels indicating a higher risk of developing SMPP. Therefore, monitoring changes in peripheral serum albumin can facilitate the early prediction of the occurrence and progression of severe *Mycoplasma pneumoniae* pneumonia.

This study has several limitations that should be acknowledged. First, it was a single-center retrospective study, which may introduce selection bias and restrict the generalizability of the findings, as regional variations in the progression of MPP) could exist. Second, external validation of our results was not performed to confirm their robustness across different populations. Thus, our conclusions require further verification through large-scale prospective multicenter studies to enhance their reliability and clinical applicability.

In conclusion, this study established a nomogram for predicting the risk of SMPP, which integrates three key indicators: duration of fever, RBC count, and ALB. This tool offers a valuable complement to existing strategies for identifying SMPP, and facilitates the timely prevention and optimized clinical management of children at risk of developing SMPP.

## Data Availability

The original contributions presented in the study are included in the article further inquiries can be directed to the corresponding authors.
